# When signals diverge, what MAUDE can and cannot tell us about subcutaneous implantable defibrillator inappropriate shocks

**DOI:** 10.1016/j.hroo.2025.11.015

**Published:** 2025-11-27

**Authors:** Christopher Monkhouse, Pier D. Lambiase, Syed Ahsan

**Affiliations:** 1 Barts Heart Centre, West Smithfield, London, United Kingdom; 2 William Harvey Research Institute, Queen Mary University of London, London, United Kingdom; 3 Institute of Cardiovascular Science, University College London, London, United Kingdom

**Keywords:** Subcutaneous ICD, Transvenous ICD, Device complications, Sudden cardiac death, Inappropriate therapy

## Introduction

We credit Hauser et al^1^ for their work using the Manufacturer and User Facility Device Experience (MAUDE) to compare inappropriate shock (IAS) reports for Boston Scientific’s EMLEM subcutaneous implantable cardioverter-defibrillator (S-ICD) with the company’s single-chamber transvenous implantable cardioverter-defibrillators (TV-ICDs) over a recent 4-year period. Their main message is simple: a disproportionate number of IASs reported involve S-ICDs, with oversensing being the dominant cause. This is potentially of concern in a field where S-ICD is now common practice and an established therapy.

At the same time, randomized and registry data have consistently suggested that S-ICDs can match TV-ICDs for overall IAS, even when compared with legacy devices.^2^ The question we should first ask is not whether S-ICDs are good or bad, but why the MAUDE data looks so unfavorable and what the community should do about it.

## Reading MAUDE against trial and registry data

MAUDE is not a denominator-based safety registry, and Hauser et al^1^ have used the best estimate to estimate prevalence. MAUDE is a spontaneous reporting system, populated mostly by manufacturers’ reports from clinicians, where the case mix is heterogeneous, duplication is possible, and narrative detail is variable. Therefore, the true incidence of events in implanted patients including IAS is significantly overinflated because only adverse events are reported. Its strength lies in surfacing the mechanism of failure and prompting quantification analysis.^3^

In the context of this comparison, this distinction is essential. Trials such as PRAETORIAN and large registries have shown that, with structured screening, implant, and programming, S-ICDs and TV-ICDs have comparable rates of IAS. Importantly, the MAUDE analysis does concur with the literature, in that IAS for AF/SVT and lead issues are not as prevalent in S-ICD as TV-ICD. This magnifies the disparity when viewed as the total counts of MAUDE reports. When estimating the IAS events per device distributed, the prevalence is 3.5% in S-ICD compared with 0.2% in TV-ICD. This incidence of IAS in TV-ICD is clearly underreported compared with any previous literature.^2^ This almost certainly reflects a combination of reporting bias (people are more likely to report S-ICD issues when they are unsure how to manage them) and the greater likelihood of clinicians to manage IAS on TV-ICD without involving the manufacturer.

In other words, we should read the MAUDE signal of where current S-ICD practice remains underdeveloped and how manufacturers and clinicians can improve.

## What were the actions after IAS events?

One unique strength of this analysis is that we can look beyond the IAS count to get a feel for what clinicians’ behavior after IAS was. Using the same MAUDE-derived dataset that underpins the paper, focusing on reports that describe an IAS or therapy (inappropriate ATP), we examined the narrative text to clarify the primary action taken for each report. We applied a simple hierarchy (device explant at the top, then vector change, therapy deactivation, nondescriptive programming change, surgical revisions, SMARTpass re-enablement, exercise testing, rescreening, medication adjustment, and finally “no action mentioned”) to assign 1 main action per report (Table 1).

**Table 1:** Primary management action after inappropriate therapy reports for EMBLEM S-ICD and transvenous ICD in MAUDE

Primary action (hierarchical)	EMBLEM S-ICD, n (%)	Transvenous ICD, n (%)
Device explanted	1577 (29.4)	147 (18.8)
Vector changed	1213 (22.6)	0 (0.0)
Therapy deactivated	271 (5.1)	21 (2.7)
Reprogrammed (unspecified)	410 (7.6)	146 (18.7)
Surgical revision/reposition	71 (1.3)	14 (1.8)
SMARTpass re-enabled	14 (0.3)	0 (0.0)
Exercise provocation test	71 (1.3)	1 (0.1)
Rescreened	6 (0.1)	0 (0.0)
Medication adjusted	3 (0.1)	3 (0.4)
No action mentioned	1726 (32.2)	449 (57.5)

EMBLEM S-ICD ≈5362 reports; transvenous ICD ≈781 reports.

ICD = implantable cardioverter-defibrillator; MAUDE = Manufacturer and User Facility Device Experience; S-ICD = subcutaneous implantable cardioverter-defibrillator.

Approximately one-third of devices were explanted, with a further 23% having a vector change programmed. In contrast, for TV-ICDs, the main action was “No action mentioned.” These findings are not perfect. They are devised from the narrative text and subject to all the limitations as discussed by Hauser et al,^1^ with an additional factor; that explanted S-ICD are likely to be reported becasue industry representetives support the explant procedure, even accounting for this the findings still tell an important story. After S-ICD IAS, clinicians seem more likely to escalate to explant as the management option. This aligns closely with previous publications and centers resorting to explant, potentially without making significant attempts to optimize the S-ICD.^4^

Perhaps most concerning is that rescreening and revision/repositioning barely register as a strategy in the S-ICD narrative. Importantly, Hauser et al^1^ noted the importance of the SMARTpass filter deactivation and the recognition of low-amplitude R-waves as early markers of future oversensing and IAS.^5^ Many of these issues can be resolved by either a vector reprogramming or a lead/generator revision. Indeed, we have previously shown success in different methods to address these issues systematically and minimize the need for S-ICD explant.^5^

## What this means for industry and the electrophysiology community

The MAUDE signal and what we see people doing after IAS can act as a pointer to where the field can improve.

For industry (across all manufacturers), the priorities suggested by these data are as follows:

• Closer postmarket surveillance of IAS across all implantable cardioverter-defibrillators. Future analysis should incorporate denominators, including all geographies and service models, to capture the real-world impact of the technologies.
• Education and practical guidance as a part of product stewardship. The pattern of frequent explant and limited rescreening suggest that troubleshooting pathways are not yet standardized, highlighting a clear opportunity for pragmatic S-ICD optimization and troubleshooting guidance from clinical experts.

For the electrophysiology community, the implications are complementary:

• Define and endorse minimum education standards for the whole journey of S-ICD patients; S-ICD screening, implant, and follow-up, comparable with what exists for TV-ICD systems.
• Develop better methods to capture complications and therapies to incorporate reporting into routine practice. In addition, cardiac device services should aspire to record simple, systematic registries or network-level databases to allow a more complete analysis in the future.

## Conclusion

The MAUDE analysis can be interpreted as showing that the S-ICD performance is highly dependent on sensing margins, operator technique, and structured follow-up. When things go wrong, real-world practice often escalates to explant, with surprisingly little optimization by re-intervention. If we take that signal seriously, the response should not be to abandon S-ICD, but rather to standardize how we implant, monitor, and troubleshoot the issues, meaning that fewer patients have IAS irrespective of the center responsible for their ongoing care.

## Declaration of generative AI and AI-assisted technologies in the manuscript preparation process

During the preparation of this work, the authors used ChatGPT 5.1 (OpenAI, San Francisco, CA) to assist with phrasing and editing for clarity. After using this tool/service, the authors reviewed and edited the content as needed and take full responsibility for the content of the published article.
